# A rare case of double orifice mitral valve with perimembranous ventricular septal defect: Application of three-dimensional echocardiography for clinical decision making

**DOI:** 10.4103/0974-2069.64362

**Published:** 2010

**Authors:** Rohit Tandon, Shibba Takkar, Shailender Kumbhkarni, Naveen Kumar, Naved Aslam, Bishav Mohan, G S Wander

**Affiliations:** Dayanand Medical College and Hospital, Unit Hero DMC Heart Institute, Tagore Nagar, Civil Lines, Ludhiana, India

**Keywords:** Double orifice mitral valve, real-time three-dimensional echocardiography

## Abstract

Double orifice mitral valve (DOMV) is an uncommon anomaly of surgical importance characterized by a mitral valve with a single fibrous annulus with two orifices opening into the left ventricle (LV). Subvalvular structures, especially the tensor apparatus, invariably show various degrees of abnormality. Associated congenital heart defects are common, though DOMV can occur as an isolated anomaly. Two-dimensional echocardiography is useful for diagnosis but combining it with real-time three-dimensional echocardiography helps in a more detailed evaluation of mitral valve and subvalvular structures as is shown in this case description.

## INTRODUCTION

Double orifice mitral valve (DOMV) was described for the first time by Greenfield in 1876. Since that time, more than 200 cases have been reported.[1] The lesion consists of two anatomically distinct orifices separated by an accessory fibrous tissue. The most common type (accounts for 85% cases) is the eccentric or hole type that is characterized by a small accessory orifice situated at one of the commissures. Other less common types are central type (accounts for 15% cases) and duplicate mitral valve.[2] Most of the cases are diagnosed by trans-thoracic (TTE) and trans-esophageal (TEE) two-dimensional echocardiography (2DE). We describe a case of DOMV with associated ventricular septal defect (VSD) with incremental role of real-time three-dimensional echocardiography (RT3DE) in its assessment.

## CASE REPORT

**Figure 1:**
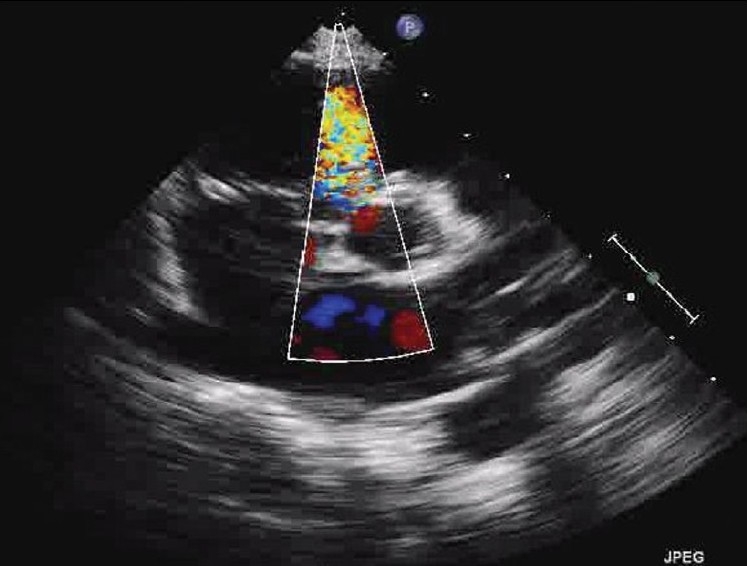
TTE image parasternal short axis view showing small perimembranous VSD with left to right shunt

**Figure 2:**
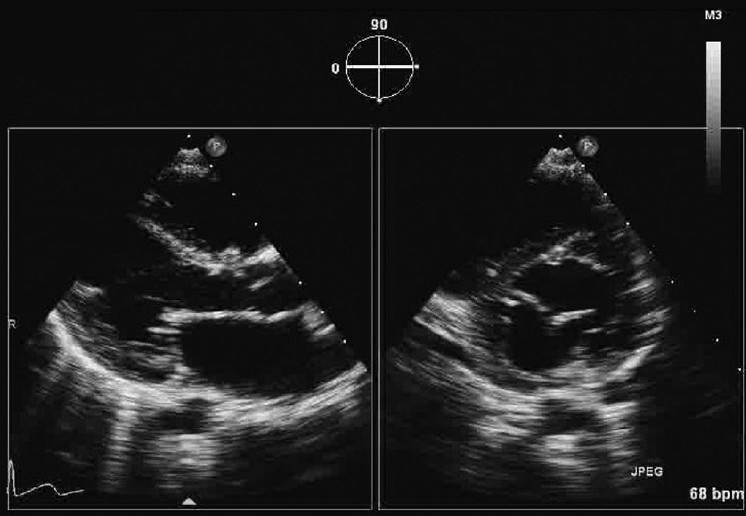
Simultaneous parasternal long axis and short axis view showing two separate mitral orifices

A five-year-old child was referred to us for evaluation of a systolic murmur and failure to thrive. General examination was unremarkable except for undernutrition. Cardiac evaluation revealed a pansystolic murmur in left paratsernal region with normal heart sounds. Chest X-ray and ECG were unremarkable. Two dimensional TTE confirmed a small perimembranous ventricular septal defect with left to right shunt [Figure 1]. Apical four chamber view showed a small tissue like structure dividing the common mitral annulus into two. Both, the septal and the lateral, annuli were guarded by two leaflets [Figure 2]. Color flow examination did not demonstrate any region of flow convergence or regurgitation Pulse Doppler flow across respective orifices showed normal mitral flow. Short axis parasternal view showed two separate mitral orifices giving a knot like appearance instead of usual fish mouth appearance with a single orifice [Figure 2]

**Figure 3:**
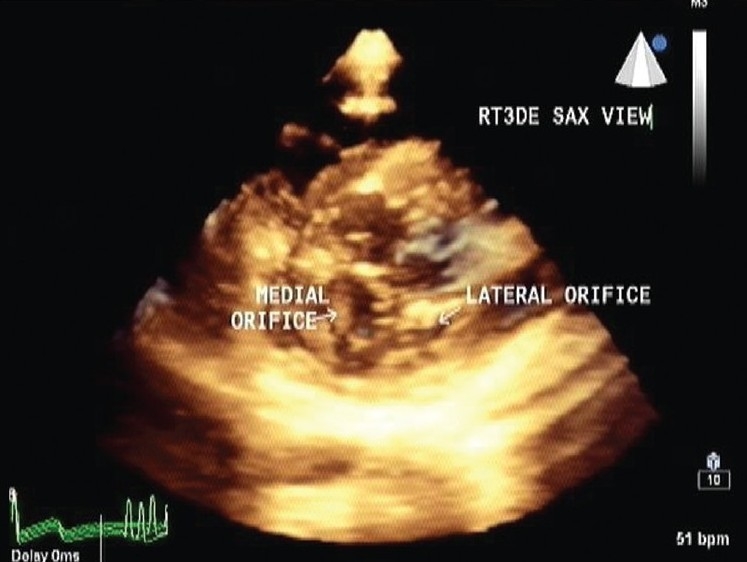
En-face atrial view showing clearly two separate mitral orifices

**Figure 4:**
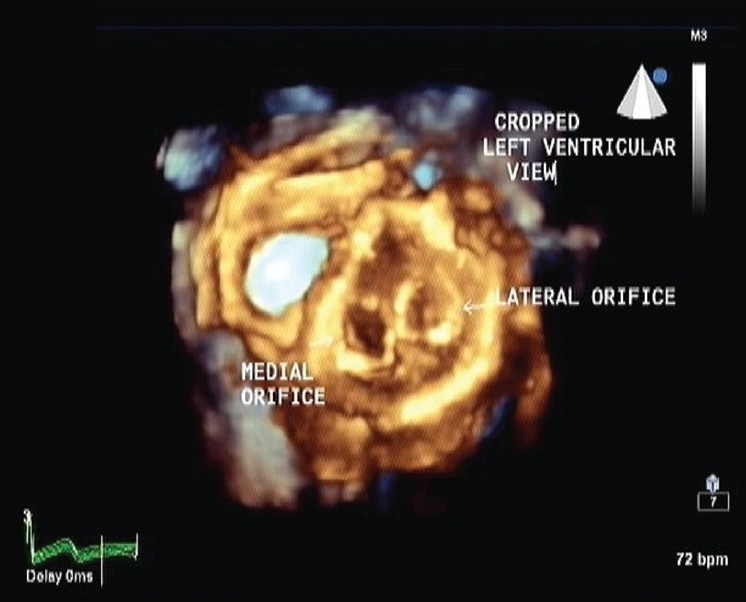
En-face ventricular view demonstrating separate chordal insertion for each orifices

RT3DE was performed in order to further evaluate this unusual mitral valve anatomy. RT3DE views from left atrial and ventricular sides confirmed the symmetry of both medial and lateral orifices, the central location of abnormal fibrous leaflet tissue, extension of fibrous tissue in ventricle, chordal and papillary muscles insertion on either side [Figures 3 and 4].

## DISCUSSION

The normal mitral valve consists of a large, central orifice located between a large sail-like anterior leaflet and a small C-shaped posterior leaflet, whereas in DOMV, abnormal tissue divides the orifice into two parts.

The following three major types of DOMV are recognized.

Eccentric or hole type: This is the most common variety of DOMV (accounting for about 85%), characterized by a small accessory orifice situated at either the anterolateral or posteromedial commissure. Other anomalies of the valve apparatus, such as cleft leaflets, accessory papillary muscles, fused papillary muscles and crossing chordae tendineae, are commonly present. When the accessory orifice is located at the posteromedial commissure, an atrioventricular septal defect (AVSD) is usually present.

Central or bridge type: In about 15% of patients with DOMV, a central bridge of fibrous or abnormal leaflet tissue connects the two leaflets of the mitral valve, dividing the orifice into medial and lateral parts. These two openings may be equal or unequal, and the papillary muscles are usually normal, with chordae surrounding each orifice inserting into one papillary muscle. In this type of DOMV, dilatation of the posteromedial orifice is feasible by means of balloon valvuloplasty.

Duplicate mitral valve: This condition involves two mitral annuli and valves, each with its own set of leaflets, commissures, chordae and papillary muscles.[2,3]

The embryologic theories explaining its origin include abnormal leaflet fusion and persistence of left part of the common atrioventricular canal. The combined area of DOMV in the presence of an AVSD is 85-90% of the normal expected area. With a near-normal valve, the area flow and remain adequate both at rest and during exercise. However, in the absence of an associated AVSD, the area may be substantially less than normal. Abnormal structure, including large bridging tissue, bulky abnormal leaflets, fused chordae or abnormal papillary muscles, reduce the effective area of the valve. Abnormalities in the leaflets include thickening, fusion, perforations, restricted movements and ruptured chordae with flail cusps. Such valves can result in clinically significant degrees of mitral incompetence.[4,5]

Associated congenital heart defects are common, though DOMV can occur as an isolated anomaly. The most common associated lesion is AVSD. Other lesions include VSD, coarctation of aorta and interrupted aortic arch.

The mitral valve is functionally normal in about 50% of patients and significant stenosis or regurgitation is present in the rest.[6]

In an asymptomatic patient, DOMV can be an incidental finding during echocardiographic examination. Though 2DE is useful in diagnosing the abnormal mitral valve anatomy, RT3DE examination provides additional anatomic details like the central bridging tissue and its extent to ventricular side and its spatial orientation to other sub-mitral structures[7,8] as was seen in this patient. Management needs to be individualized based on the associated lesions, extent of physiological derangement (stenosis or regurgitation) and clinical symptoms.
